# Plexiform Unicystic Ameloblastoma: A Rare Variant of Ameloblastoma

**DOI:** 10.1155/2014/146989

**Published:** 2014-08-14

**Authors:** Swapnil S. Deore, Rishikesh C. Dandekar, Aarti M. Mahajan, Rahul Patil, Nilima Prakash

**Affiliations:** ^1^Department of Oral Pathology & Microbiology, Jawahar Medical Foundation's Annasaheb Chudaman Patil Memorial Dental College & Hospital, Sakri Road, Morane, Dhule, Maharashtra 424001, India; ^2^Department of Oral Pathology & Microbiology, Mahatma Gandhi Vidyamandir's Karmaveer Bhausaheb Hiray Dental College & Hospital, Nashik, Maharashtra 422003, India

## Abstract

The term plexiform unicystic ameloblastoma refers to a pattern of epithelial proliferation that has been described in cystic cavity. Because of unilocular presentation, it is commonly misdiagnosed as an odontogenic cyst. However, they may often behave clinically as biologically aggressive tumors. These tumors show high incidence of cortical perforation, tooth resorption and a high rate of recurrence after simple enucleation. This paper aims to provide an insight into this biologically distinct entity. A literature review on the topic has been added along with a case report highlighting the approach of diagnosis and management of such ameloblastomas.

## 1. Introduction

Ameloblastoma is the most common odontogenic neoplasm. Churchill is credited with the first use of the term ameloblastoma in 1934 [1]. A thorough description of ameloblastoma was given by Falkson in 1879, and since then, thousands of reports on ameloblastoma have been published [1]. There are four subtypes or variants of ameloblastomas which can presently be distinguished:

- the classic solid/multicystic ameloblastoma (SMA),
- the unicystic ameloblastoma (UA),
- the peripheral ameloblastoma (PA),
- the desmoplastic ameloblastoma (DA), including the so-called hybrid lesions.

The relative frequency of unicystic ameloblastoma has been reported as 5% and 22%. Robinson and Martinez in 1977 were the first to describe unicystic ameloblastoma and to call for recognition of the entity [2]. Plexiform unicystic ameloblastoma is a relatively rare variant of unicystic ameloblastoma. We report a case of plexiform unicystic ameloblastoma of mandible in a 15-year-old male.

## 2. Case Report

A 15-year-old male patient reported with the chief complaint of swelling on the left side of lower jaw since 9-10 months. He noticed a swelling in the lower jaw in the posterior region, which was initially small in size and gradually increased to the present size. It was initially painless, but the patient now complains of mild intermittent pain, occasionally. The patient was prescribed antibiotics and analgesics by a general practitioner 3-4 times in the last 9 months.

**Figure 1:**
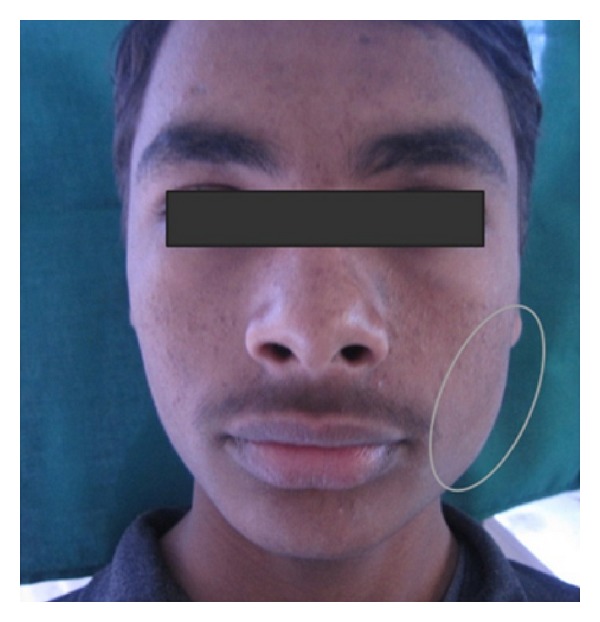
Diffuse swelling at the angle of left side of mandible.

Extraoral examination revealed a solitary swelling in the left mandibular ramus area. The swelling was roughly oval in shape with approximate size of 2 cm × 3 cm. The margins of the swelling were diffuse. The skin overlying the swelling was smooth and normal in color (Figure 1).

On palpation, temperature of overlying skin of swelling was slightly elevated. The consistency of the swelling was bony hard. Medio-lateral expansion of the cortical plates was noted at angle of mandible. A single left submandibular lymph node of size approximately 1 to 1.5 cm was noted, which was slightly tender and mobile.

**Figure 2:**
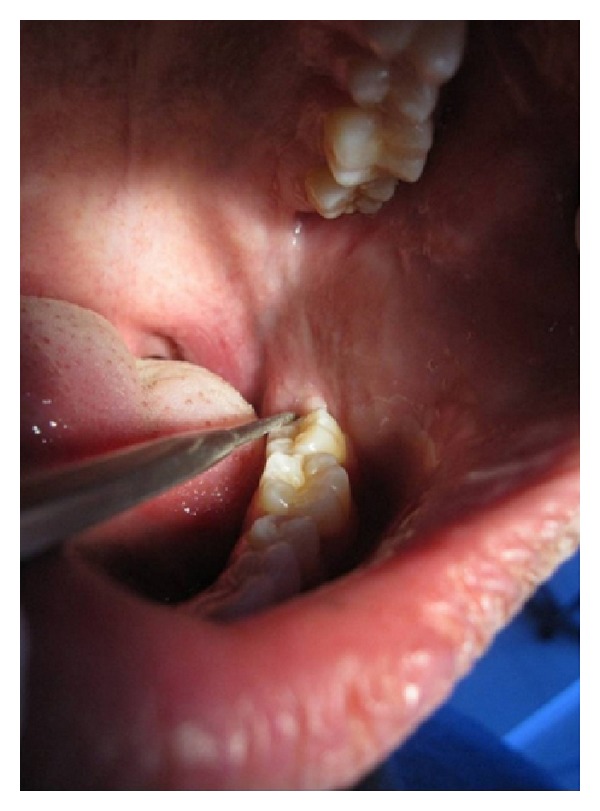
Slightly obliterating the pterygopalatine raphe and a deep periodontal pocket was noted distal to 37.

Intraoral examination revealed a single small swelling in retromolar area, slightly obliterating the pterygopalatine raphe. Expansion of buccal and lingual cortical plate was noted. A deep periodontal pocket was noted distal to 37 (Figure 2).

Provisional diagnosis on clinical examination was made as benign odontogenic lesion. Differential diagnosis of ameloblastoma, odontogenic keratocyst, dentigerous cyst associated with 38, calcifying odontogenic cyst, calcifying epithelial odontogenic tumor, and ameloblastic fibroma was considered.

**Figure 3:**
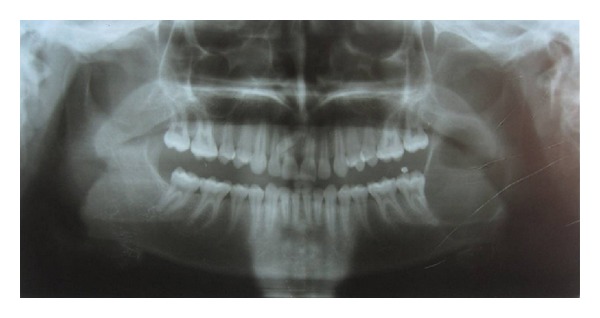
Unilocular radiolucency involving the ramus of left side of mandible.

Radiographic examination of the lesion showed a well-defined unilocular radiolucency involving the left side of the mandible which extended anteroposteriorly from the distal surface of the left mandibular second molar to the posterior border of ramus of mandible and superoinferiorly from coronoid notch to the inferior border of mandible. It showed well corticated borders. Resorption of the distal surface of root of the mandibular second molar was also noted (Figure 3).

**Figure 4:**
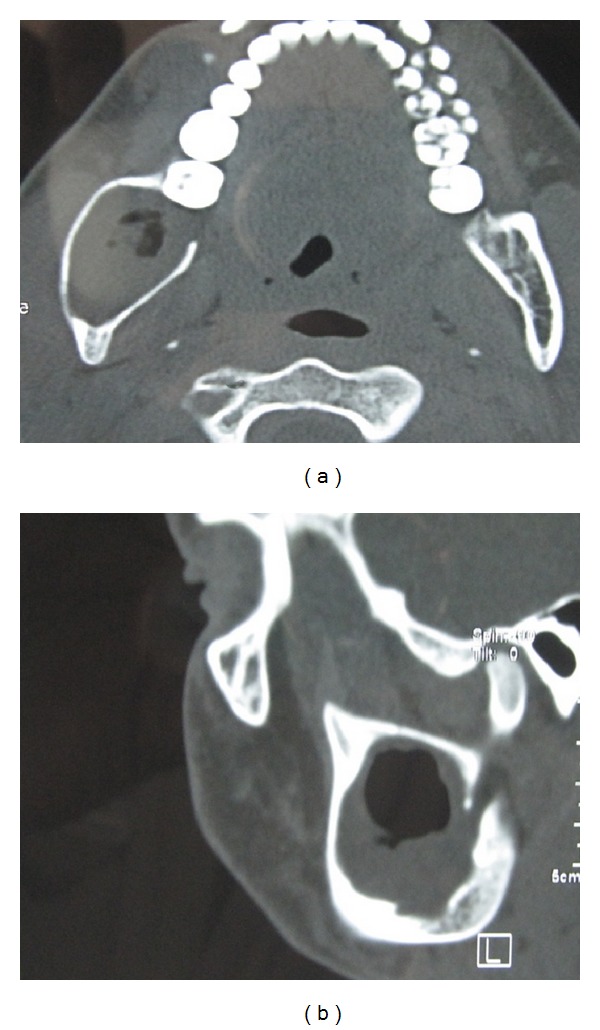
CT scan of the lesion showing a unilocular osteolytic lesion with posterior part of body and ramus of the left side of mandible revealing bilateral cortical plate expansion and perforation of the lingual cortical plate.

**Figure 5:**
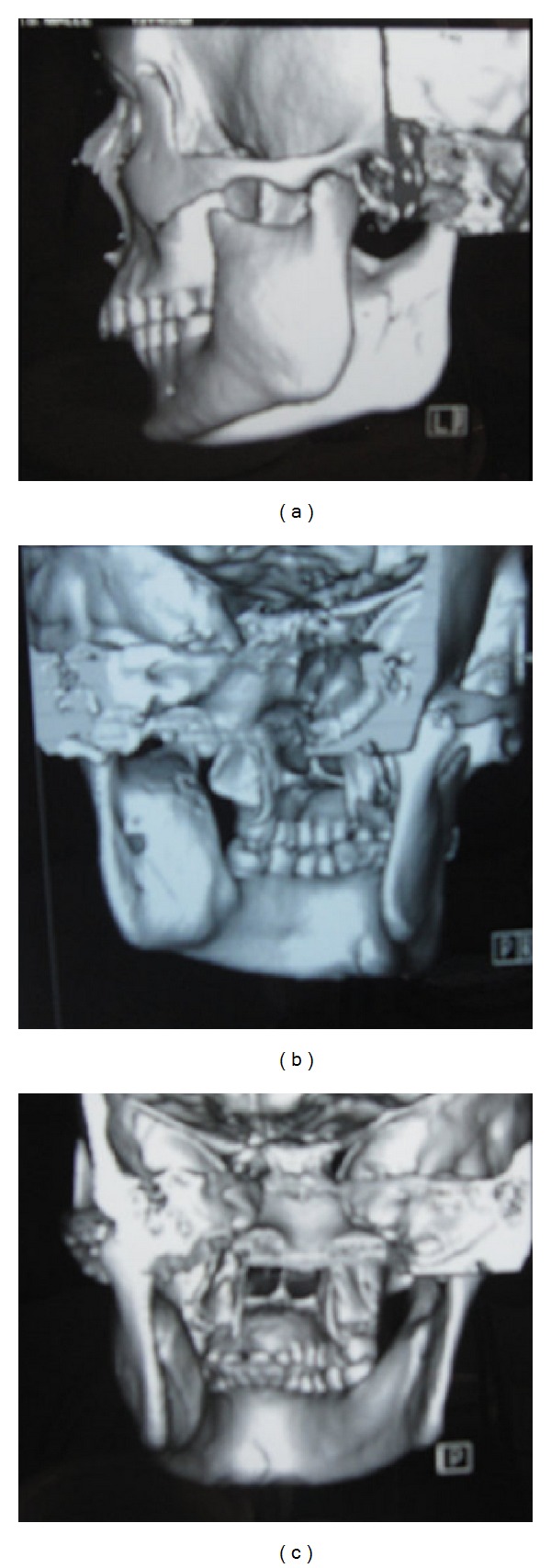
3D CT scan of the lesion showing bilaterally expansile lesion with posterior part of body and ramus of the left side of mandible.

CT and 3D CT scan of the lesion showed a unilocular osteolytic lesion in the posterior part of body and ramus of the mandible. Bilateral cortical plate expansion was noted. Perforation of the lingual cortical plate was also revealed (Figures 4 and 5). Radiologic examination showed that mandibular left third molar was absent. So the possibility of “dentigerous cyst associated with 38” which was considered in the provisional diagnosis was ruled out.

**Figure 6:**
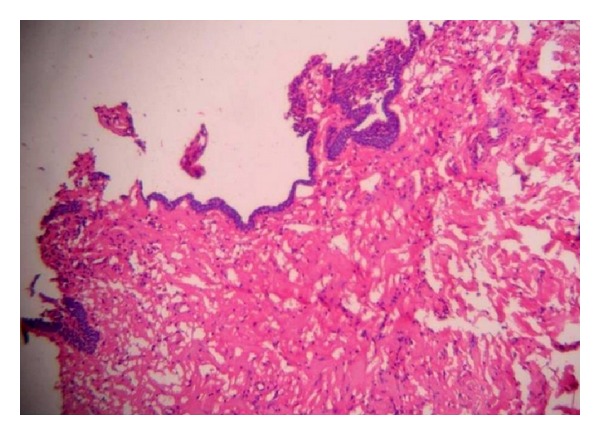
Cystic cavity lined by odontogenic epithelium and connective tissue capsule.

Routine hemogram was performed and all the blood indices were within normal limits. An incisional biopsy was then performed, which on histopathologic examination revealed a cystic cavity lined by odontogenic epithelium and a connective tissue capsule. Epithelium shows palisaded basal layer resembling ameloblast-like cells and a superficial layer showing stellate reticulum-like cells. The connective tissue was dense, fibrous with collagen fibers arranged haphazardly. Numerous engorged and dilated blood vessels were seen (Figure 6).

**Figure 7:**
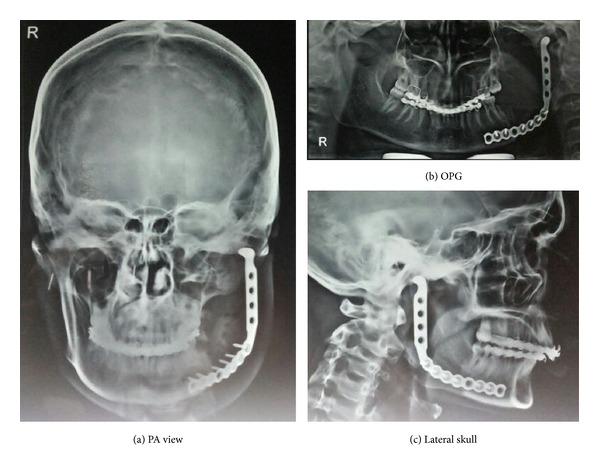
Postoperative radiographs: (a) PA view, (b) OPG, and (c) lateral skull.

From the above clinicopathological features, a diagnosis of unicystic ameloblastoma was made. The patient then underwent a mandibular segmental resection involving condyle and reconstruction was done with 2.7 mm titanium reconstruction plate and iliac crest graft, under general anesthesia. Healing was uneventful. Patient was followed up after 1 month with radiographic evaluation which showed complete healing of wounds and well maintained graft (Figure 7).

**Figure 9:**
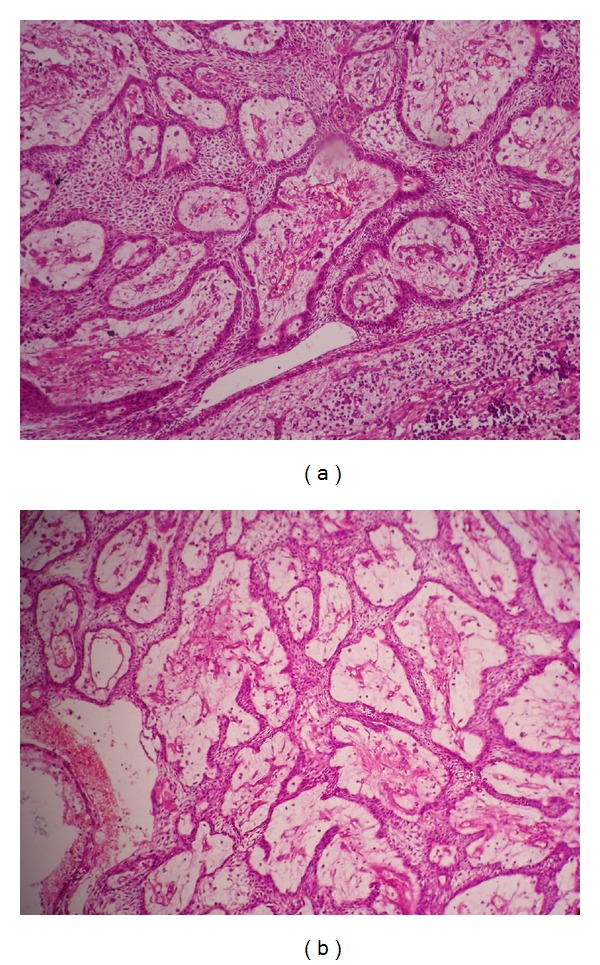
H & E stained section showing ameloblastic cystic epithelium showing intraluminal proliferation in the form of plexiform pattern ((a) H & E stain; 100x and (b) H & E Stain; 40x).

We received a segmental resected specimen involving condyle, coronoid process, upto ascending ramus, which showed perforation in the anterior border of ascending ramus. Histopathological examination of the excisional biopsy specimen showed lesional tissue that consisted of a cystic cavity lined by odontogenic epithelium and connective tissue capsule. The epithelium showed cuboidal or columnar basal cells with hyperchromatic nuclei, nuclear palisading with polarization, cytoplasmic vacuolization with intercellular spacing, and subepithelial hyalinization and superficial layer showing stellate reticulum-like cells. There was also proliferation of these cells in cystic lumen in a plexiform pattern. The cells are arranged in interconnecting strands and cords with peripheral palisaded layer and central stellate reticulum-like cells (Figure 9). Tissue material resembling an odontogenic keratocyst lining was not observed, even with serial sections of tissue. So possibility of ameloblastic transformation of odontogenic keratocyst was also excluded.

According to the classification suggested by Ackermann et al. [3], it was classified as unicystic ameloblastoma subgroup 1.2 which is also known as plexiform unicystic ameloblastoma.

## 3. Discussion

The unicystic ameloblastoma, a variant of ameloblastoma, is reported to have less aggressive biologic behavior and lower recurrence rate than the classic solid or multicystic ameloblastoma. Although the unicystic ameloblastoma is a “cystic” appearing lesion on gross examination, subsequent microscopic examination shows the presence of an ameloblastoma within the cyst wall. Prior to the report by Robinson and Martinez, this variant had been referred to as a mural or intraluminal ameloblastoma. The minimum criterion for diagnosing a lesion as UA is the demonstration of a single (often macro-) cystic sac, with an odontogenic (ameloblastomatous) epithelium, which is usually present only in focal areas.

The histologic features of UA have been established by several authors, all of whom recognize various subtypes. The most accepted histologic classification of UA is that suggested by Ackermann et al. [3] who classified it into following four histologic subgroups:

 (1) luminal UA;
 (1.2) luminal and intraluminal UA;
 (1.2.3) luminal, intraluminal, and intramural UA;
 (1.3) luminal and intramural UA.

The luminal type of tumor is called UA subgroup (1) which is defined as a cystic cavity lined by an epithelial lining of which parts show transformation to cuboidal or columnar basal cells with hyperchromatic nuclei, nuclear palisading with polarization, cytoplasmic vacuolization with intercellular spacing, and subepithelial hyalinization. This definition was originally suggested by Vickers and Gorlin [4].

UA subgroup (1.2) shows simple and intraluminal features. The intraluminal proliferation of ameloblastic epithelium is in the form of plexiform pattern. Hence, this subgroup is sometimes referred to as the plexiform unicystic ameloblastoma.

UA subgroup (1.2.3) covers cases where there is an occurrence of intramural ameloblastoma tissue as well as subgroup (1.2) features.

The last subgroup (1.3) exhibits a cyst with a luminal lining in combination with intramural nodules of SMA tissue. It is important to stress that these four subgroups occur in both the dentigerous and the nondentigerous variants.

The present case shows presence of plexiform ameloblastoma in continuity with the cyst lining proliferating into the cystic lumen and hence was diagnosed as subgroup (1.2) plexiform unicystic ameloblastoma.

Philipsen and Reichart [5] in their critical review of 193 cases of UA divided the material into two categories: histologically verified UAs associated with an unerupted tooth and UAs lacking an association with an unerupted tooth. The present case was not associated with any unerupted tooth, so this tumor can be termed as nondentigerous variant.

The cases diagnosed as dentigerous (*n* ~ 23) occurred in much younger patients (mean 16.5 years) than those diagnosed as nondentigerous (*n* ~ 17; mean 35.2 years) [5]. In contrast to this finding, the present case was of nondentigerous type, but it has occurred in 2nd decade of life. Compared to multicystic ameloblastoma, unicystic variety occurs more commonly at younger age as in our patient.

In regard to gender distribution, the UA dentigerous variant shows a slight male predominance with a male : female ratio of 1.6 : 1. However, when the tumor is not associated with an unerupted tooth, the gender ratio is reversed to a male : female ratio of 1 : 1.8 [5]. Hence, though the nondentigerous variant is seen more commonly in females, it was not so in the present case which was seen in a male patient.

The location of the UA within the jawbones shows marked predominance of the mandible irrespective of the variant. The posterior mandible, including the ascending ramus, is the region most often affected in both variants [5]. In the present case too, the ramus was primarily involved.

The radiographic appearance of all UAs is divided into the two main patterns, unilocular and multilocular; there is clear predominance of the unilocular configuration in all studies where this feature was evaluated. This predominance was exceptionally marked for the dentigerous variant where the unilocular: multilocular ratio was 4.3 : 1 [6]. For the nondentigerous type this ratio was 1.1 : 1.

Leider et al. [7] proposed three pathogenic mechanisms for the evolution of UA: (1) the reduced enamel epithelium associated with a developing tooth undergoes ameloblastic transformation with subsequent cystic development; (2) ameloblastomas arise in dentigerous or other types of odontogenic cysts in which the neoplastic ameloblastic epithelium is preceded temporarily by a nonneoplastic stratified squamous epithelial lining; and (3) a solid ameloblastoma undergoes cystic degeneration of ameloblastic islands with subsequent fusion of multiple microcysts and develops into a unicystic lesion.

Li et al. [8] found that all areas of UA lining contained significantly more PCNA positive cells than dentigerous cyst linings even in areas where epithelial morphology was similar to that of the dentigerous cyst lining. This finding favoured the concept that UAs are de novo cystic neoplasms.

Li et al. [8] did not find a true dentigerous arrangement in any of their seven cases of the dentigerous variant. This finding was interpreted as an argument against the hypothesis that UA may originate from a preexisting dentigerous cyst. Similar observations were made by Philipsen et al. [9] when they examined the dentigerous appearance characteristic of another odontogenic tumor, the follicular variant of the adenomatoid odontogenic tumor (AOT). The lack of a true dentigerous cyst-impacted tooth relationship did not support the AOT originating from a preexisting dentigerous cyst but rather favored the “envelopmental” concept, that is, an unerupted tooth being embedded in an expanding tumor mass, whether cystic or solid.

Immunocytochemical markers for lectins (*Ulex europaeus agglutinin I* and* Bandeiraea simplicifolia agglutinin I*) and proliferating cells (proliferating cell nuclear antigen and Ki-67) may be helpful in differentiating UA from any other cyst [8, 10, 11]. Studies should be conducted to find whether the intraluminal exophytic masses (subgroup 1.2 or plexiform UA) are truly tumorous proliferation or just only represent a nonneoplastic, plexiform epithelial hyperplasia.

Various treatment modalities for plexiform unicystic ameloblastoma have been used such as enucleation, enucleation followed by application of Carnoy's solution, marsupialization followed by surgery, and segmental resection. The recurrence rate after enucleation alone is the highest (30.5%), while resection of PUA results in the lowest recurrence rate (3.6%) [12]. In our case, segmental resection was carried out because of the extensive size of the lesion.

## 4. Conclusion

In most cases, unilocular lesions are diagnosed as odontogenic cyst both clinically and radiographically. Hence the chances of treating the lesion conservatively are more. Enucleation or excisional biopsy is the most preferred and planned treatment in case of odontogenic cysts. An accurate and timely diagnosis of the character and extent of unicystic ameloblastoma should be done which is only possible after a thorough microscopic examination of the lesion. We would like to emphasize the importance of the microscopic examination of all lesions mimicking odontogenic cyst prior to the treatment plan. Adequate radical resection of unicystic ameloblastomas is important to avoid further complications and recurrence.

## Figures and Tables

**Figure 8:**
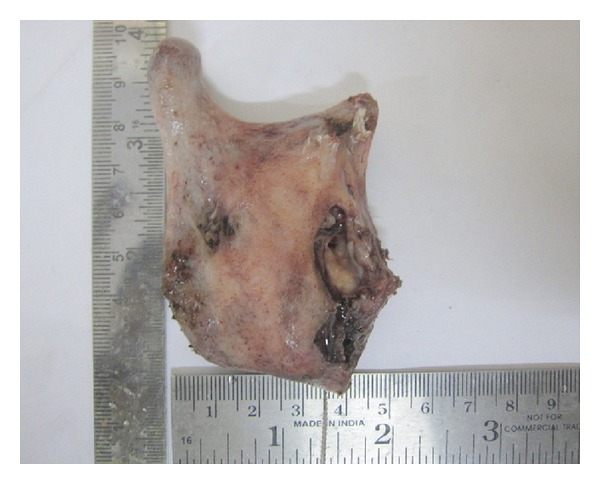
Resected specimen of mandible showing perforation at the anterior border of mandible.
